# Effectiveness of Antenatal Interventions in Preventing Pregnancy-Related Complications: A Systematic Review

**DOI:** 10.7759/cureus.116832

**Published:** 2026-09-24

**Authors:** Uppalapati Priyanka, Pranjal Upadhayay, Bikash Majumder, Sumit Sukla Das, Narayan Kamath, Darshna Sarvaiya, Nikeeta Ashokrao Khanorkar

**Affiliations:** 1 Department of Obstetrics and Gynaecology, All India Institute of Medical Sciences (AIIMS), Mangalagiri, Mangalagiri, IND; 2 Department of Public Health and Medical Education, National Medical College and Teaching Hospital, Birgunj, NPL; 3 Department of Obstetrics and Gynaecology, Agartala Government Medical College, Agartala, IND; 4 Department of Microbiology, NAMO Medical Education and Research Institute and NAMO Hospital, Silvassa, IND; 5 Department of Community Medicine, Dr. N.D. Desai Faculty of Medical Science and Research, Dharmsinh Desai University, Nadiad, IND; 6 Department of Obstetrics and Gynaecology, Government Medical College, Nagpur, Nagpur, IND; 7 Department of Obstetrics and Gynaecology, Maharashtra University of Health Sciences, Nashik, IND

**Keywords:** calcium supplementation, gestational diabetes mellitus, lifestyle interventions, preeclampsia, pregnancy outcomes

## Abstract

Gestational diabetes mellitus, preeclampsia, and other adverse pregnancy outcomes continue to contribute substantially to maternal and neonatal morbidity worldwide. The increasing burden of metabolic and hypertensive complications during pregnancy has strengthened the need for effective, targeted preventive strategies. Although pharmacological, lifestyle, dietary, and healthcare system interventions have been widely studied, their effects vary according to the clinical outcome, population risk profile, intervention intensity, timing, adherence, and healthcare setting. Gaps therefore remain in identifying which approaches provide consistent benefit across different maternal and neonatal outcomes. This review synthesized evidence from randomized controlled trials evaluating interventions intended to improve pregnancy-related outcomes. A structured systematic search and study-selection process was undertaken, and eligible trials included pharmacological, lifestyle, dietary, behavioral, and healthcare system interventions. Data were synthesized qualitatively, and methodological quality and risk of bias were assessed using established criteria. The findings indicate that pharmacological interventions, including aspirin, calcium supplementation, and antenatal corticosteroids, showed benefits for selected outcomes in appropriately defined clinical populations and settings. Lifestyle and dietary interventions showed heterogeneous findings, with structured and intensive approaches demonstrating favorable outcomes in some populations, whereas lower-intensity interventions produced less consistent effects on major clinical endpoints. Behavioral and healthcare system interventions improved selected aspects of care delivery and patient-centered outcomes, although effects on major maternal and neonatal clinical outcomes were less consistent. Overall, the evidence supports targeted, context-specific use of preventive interventions rather than direct ranking of intervention categories, while recognizing substantial heterogeneity across populations, interventions, and outcomes.

## Introduction and background

Gestational diabetes mellitus and hypertensive disorders of pregnancy are significant causes of maternal and neonatal morbidity throughout the globe [1]. The incidence of gestational diabetes has been on the rise in the past decades around the world owing to the increased rates of obesity, older maternal age, and sedentary lifestyles [2]. There is still a high risk of preeclampsia and associated hypertensive disorders, such as preterm birth, fetal growth retardation, and cardiovascular complications in the long term in the mother and fetus [3]. The conditions constitute a major burden on the health of the population, especially in low- and middle-income environments where preventive care and early interventions are not yet available [4]. The existing prevention measures include pharmacological, nutritional, behavioral, and healthcare system-based measures. Low-dose aspirin and calcium supplementation are examples of pharmacological interventions that have been extensively studied in the prevention of preeclampsia, and evidence has shown that, in high-risk patient populations, the incidence may be reduced by the intervention [5]. Antenatal corticosteroids have also been found to be effective in enhancing the survival of neonates in the situation of an imminent preterm birth, especially when such practice is carried out in sufficiently resourced health systems. These interventions have developed a role in the clinical guidelines and have been backed by consistent results in randomized controlled trials [6].

Simultaneously, lifestyle and dietary interventions have become a growing focus of interest as possible measures to reduce metabolic complications during pregnancy. Better glycemic control has been associated with changes in eating habits, including the introduction of unsaturated fats, fibre and vegetarian foods [7]. There are structured lifestyle interventions that include dietary counselling, physical activity, and weight management and have been investigated particularly in high-risk populations that are susceptible to gestational diabetes [7]. Despite these attempts, the impact of lifestyle interventions is still not uniform, and some of the trials have shown a small or no significant decrease in primary clinical outcomes. These inconclusive outcomes are attributed to differences in intervention intensity, adherence, and timing [8]. They have also established behavioral and system-level interventions to enhance delivery of maternity care [9]. Models such as the midwifery continuity of care are designed to increase coordination, patient involvement, and the quality of care in general [10]. These techniques have demonstrated favorable results in patient experience and part of the intermediate results, including breastfeeding behaviors [11]. Nevertheless, the question remains how these improvements are translated into clinical outcomes (a reduction in the number of preterm births or metabolic complications). This gap brings out the complex nature of pregnancy-related conditions, which are identified with multifactorial biological, behavioral, and social determinants [12].

Considerable heterogeneity exists across trials evaluating preventive interventions in pregnancy because the interventions address distinct clinical targets and operate through different mechanisms. Differences in population characteristics and intervention responses may contribute to variation in outcomes across studies [13]. Pharmacological strategies such as low-dose aspirin and calcium supplementation primarily aim to prevent preeclampsia, whereas dietary and lifestyle interventions predominantly target gestational diabetes mellitus, excessive gestational weight gain, and related metabolic complications. Antenatal corticosteroids are used in women at risk of imminent preterm birth to reduce neonatal morbidity and mortality, while healthcare system interventions primarily seek to improve care coordination, access, continuity, and patient-centred maternity care. These intervention categories therefore should not be considered directly interchangeable, and their effectiveness must be interpreted according to the clinical outcome they are designed to influence.

Important uncertainties also remain regarding the timing, intensity, adherence, and clinical setting of these interventions. Some preventive strategies are initiated during pregnancy, whereas others may begin before conception in women at increased risk of pregnancy-related complications [14]. Differences in baseline risk, healthcare infrastructure, intervention intensity, and participant adherence may further influence observed treatment effects, particularly because sustained adherence to behavioral interventions can be difficult to maintain over time [15]. A systematic synthesis that evaluates these interventions within their respective clinical targets is therefore needed to determine where evidence of benefit is most consistent and where uncertainty remains.

Objectives of the review

This systematic review aims to evaluate randomized controlled trials of preconception and antenatal pharmacological, dietary, lifestyle, behavioral, and healthcare system interventions designed to prevent or reduce pregnancy-related maternal and neonatal complications. The review specifically examines intervention effects on preeclampsia and other hypertensive disorders, gestational diabetes mellitus and related metabolic outcomes, preterm birth-related neonatal outcomes, and selected healthcare-delivery outcomes. The review also assesses how intervention timing, intensity, adherence, population risk profile, and healthcare setting may influence effectiveness.

PICO framework

The review question was structured according to the Population, Intervention, Comparator, and Outcome (PICO) framework. The population included pregnant women and women planning pregnancy who were eligible for preventive interventions. The interventions comprised pharmacological, dietary, lifestyle, behavioral, and healthcare system-based strategies initiated either before conception or during pregnancy. The comparators included placebo, usual or standard care, alternative doses, or other active interventions, depending on the individual trial. The outcomes were assessed according to the clinical target of each intervention and included preeclampsia and other hypertensive disorders of pregnancy, gestational diabetes mellitus and related metabolic outcomes, preterm birth, neonatal mortality, gestational weight gain, glycemic measures, neonatal growth outcomes, breastfeeding-related outcomes, and measures of maternity-care delivery. Only randomized controlled trials were included.

## Review

Methodology

Search Strategy

A systematic literature search was conducted in PubMed/MEDLINE, Scopus, Web of Science, and Cochrane CENTRAL to identify randomized controlled trials evaluating preconception and antenatal interventions for preventing or reducing pregnancy-related maternal and neonatal complications. The search covered studies published from January 2015 to July 2026, with the final database search completed in July 2026. Controlled vocabulary terms, including Medical Subject Headings (MeSH) where applicable, were combined with free-text terms using the Boolean operators AND and OR. Search concepts included pregnancy, preconception, antenatal care, gestational diabetes mellitus, preeclampsia, preterm birth, aspirin, calcium supplementation, antenatal corticosteroids, dietary intervention, lifestyle intervention, physical activity, and healthcare-system intervention. Reference lists of eligible studies and relevant reviews were additionally screened to identify potentially eligible randomized trials. The study-selection process followed Preferred Reporting Items for Systematic Reviews and Meta-Analyses (PRISMA) 2020 recommendations [16]. The databases searched, corresponding search periods, and final search dates are summarized in Table 1.

**Table 1:** Database Search Period and Final Search Dates

Database	Search period	Date last searched
PubMed/MEDLINE	January 2015–July 2026	July 12, 2026
Scopus	January 2015–July 2026	July 14, 2026
Web of Science	January 2015–July 2026	July 14, 2026
Cochrane CENTRAL	January 2015–July 2026	July 15, 2026

*Study Selection and Screening* After removal of duplicate records, two reviewers independently screened the titles and abstracts against the predefined eligibility criteria. Potentially eligible articles were then evaluated independently by the same two reviewers at the full-text stage. Disagreements at either screening stage were resolved through discussion and consensus; when consensus could not be reached, a third reviewer adjudicated the decision. Reasons for exclusion at the full-text stage were documented and incorporated into the PRISMA flow diagram. Screening was therefore performed independently and in duplicate.

Eligibility Criteria

Inclusion criteria: Eligible studies were randomized controlled trials involving pregnant women or women planning pregnancy in whom a preventive intervention was initiated during the preconception or antenatal period. Eligible interventions included pharmacological, dietary, lifestyle, behavioral, and healthcare system-based strategies intended to prevent or reduce maternal or neonatal complications. Comparators included placebo, usual care, alternative doses, or other active interventions. Studies were required to report at least one clinically relevant maternal, neonatal, metabolic, or healthcare-delivery outcome, including preeclampsia, gestational diabetes mellitus, gestational weight gain, preterm birth, neonatal mortality, or related outcomes. Only peer-reviewed articles published in English were included.

Exclusion criteria: The exclusion criteria were case reports, editorials, and observational studies. Studies that were not linked to the population with pregnancy issues or did not describe any relevant maternal or newborn outcomes were excluded. Poorly described studies in terms of their methodology, those published in other languages, and those that were not found were also filtered out. The duplicated publications and studies whose data were not clear or complete were filtered out during the screening.

Data Extraction and Analysis

Data were extracted using a standardized data-extraction framework. Two reviewers independently extracted study design and setting, participant characteristics, sample size, intervention and comparator details, primary and secondary outcomes, effect estimates, confidence intervals, P values, and relevant contextual findings. Where the source article reported an effect measure and 95% confidence interval, both were extracted and presented. When an effect estimate was not available, event proportions, mean differences, or P values were reported as available rather than replacing the findings with qualitative descriptors. Extracted data were cross-checked for accuracy, and disagreements were resolved through discussion and consensus. Owing to substantial clinical and methodological heterogeneity in populations, interventions, comparators, and outcomes, quantitative pooling was not considered appropriate. A structured synthesis without meta-analysis was undertaken. A structured synthesis without meta-analysis was undertaken. Studies were grouped according to intervention category and clinical outcome. Within each group, the direction and magnitude of effects, precision of estimates, population and intervention characteristics, and risk-of-bias judgments were considered together. Findings from studies with greater methodological concerns were interpreted more cautiously, and no indirect quantitative comparisons between intervention categories were performed.

Risk of Bias Assessment

Risk of bias in the included randomized controlled trials was assessed using the Cochrane Risk of Bias 2 (RoB 2) tool [17]. Two reviewers independently assessed each study across the five RoB 2 domains: bias arising from the randomization process, bias due to deviations from intended interventions, bias due to missing outcome data, bias in measurement of the outcome, and bias in selection of the reported result. Domain-level signaling questions and the RoB 2 decision algorithms were used to inform each judgment. Disagreements were resolved through discussion and consensus. Judgments were reported using the standard RoB 2 categories of “Low risk,” “Some concerns,” and “High risk.” Overall risk-of-bias judgments were derived according to the RoB 2 algorithm rather than by assigning numerical scores or averaging domain-level assessments.

Results

Study Selection

A total of 260 records were found in the systematic search. One hundred eighty records were screened, with 110 removed for not meeting the inclusion criteria. Eighty duplicates were removed. For the purpose of the assessment, 70 articles were assessed, and 59 articles were excluded for predefined reasons. Finally, 11 studies were selected for the review as indicated in Figure 1.

**Figure 1:**
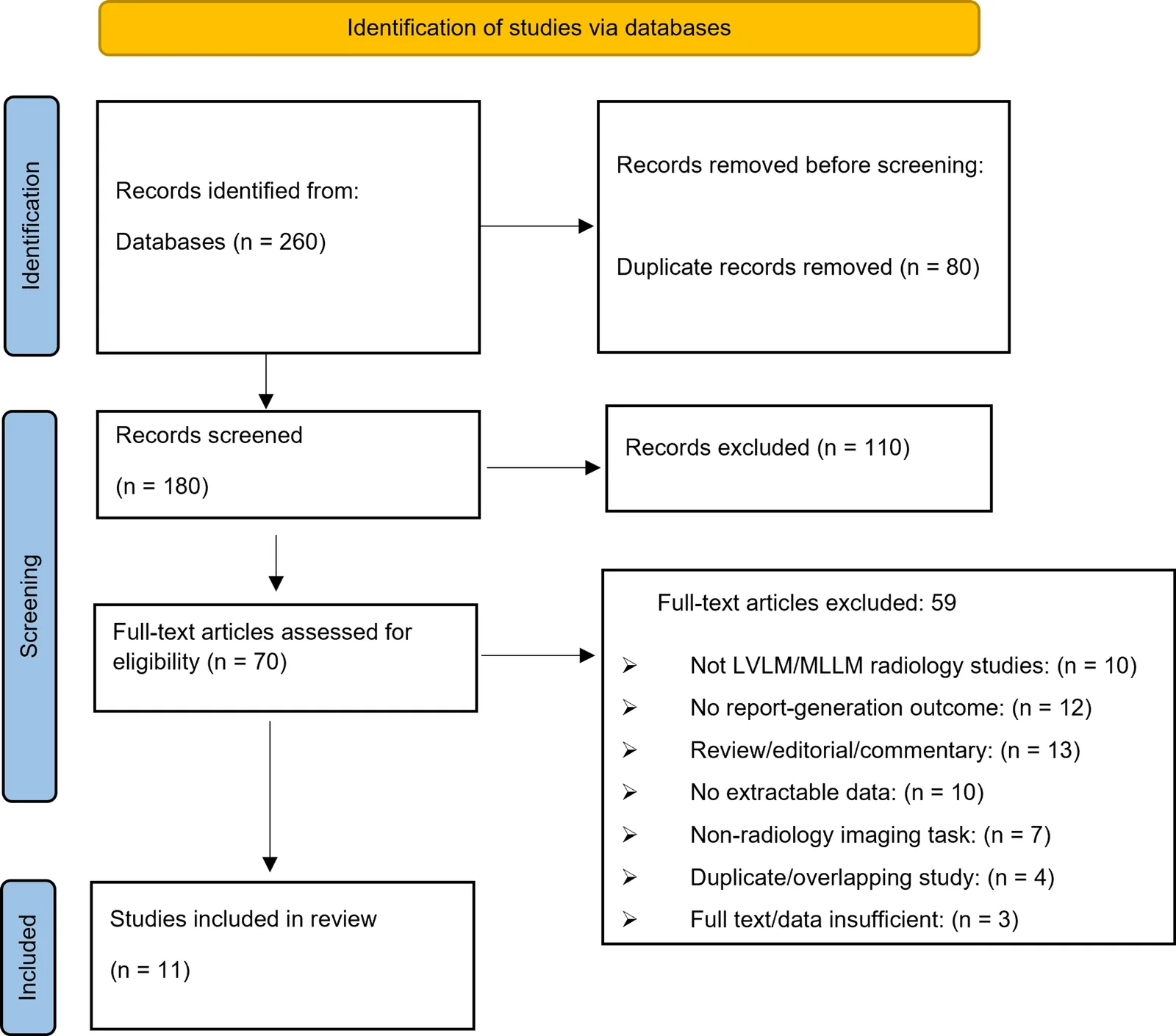
PRISMA Flow Chart PRISMA: Preferred Reporting Items for Systematic Reviews and Meta-Analyses

Study Characteristics

The studies in the included sample were randomized controlled trials that were carried out in various countries and care settings. Both general pregnant women and high-risk groups, including obese women and women who have had a history of complications associated with pregnancy, were used as the study populations. The types of interventions were diverse, such as pharmacological, diet, lifestyle, and healthcare delivery models. The most common outcomes examined included gestational diabetes mellitus, preeclampsia, preterm birth, and neonatal outcomes. The variety in the study design, the type of intervention, and population characteristics enabled a thorough comparison of the methods of improving maternal and neonatal health outcomes. The main features and results of the studies included are described in Table 2.

**Table 2:** Summary of Included Randomized Controlled Trials and Their Clinical Outcomes OR: Odds Ratio; GDM: Gestational Diabetes Mellitus; GWG: Gestational Weight Gain; PA: Physical Activity; PIH: Pregnancy-Induced Hypertension; TRE: Time-Restricted Eating; LGA: Large for Gestational Age; HE: Healthy Eating

Study	Study design & setting	Population (key characteristics)	Intervention vs comparator	Primary outcome(s)	Key results (with statistics)	Secondary findings/nuances	Interpretation/Clinical Implications
Rolnik et al. [18]	Multicenter, double-blind RCT across Europe	1776 singleton pregnancies screened as high risk for preterm preeclampsia	Aspirin 150 mg daily (11–14 to 36 weeks) vs placebo	Preterm preeclampsia (<37 weeks)	1.6% vs 4.3%; OR 0.38 (95% CI 0.20–0.74), P=0.004	No increase in maternal/neonatal adverse events; no effect on term PE	Supports a preventive benefit of aspirin when initiated early in appropriately selected high-risk pregnancies.
Dwarkanath et al. [19]	Two parallel noninferiority RCTs (India, Tanzania)	~22,000 nulliparous women combined	500 mg/day vs 1500 mg/day calcium	Preeclampsia, preterm birth	Preeclampsia: India, RR 0.84 (95% CI 0.68–1.03); Tanzania, RR 1.10 (95% CI 0.88–1.36); noninferiority met in both. Preterm birth: India, RR 0.89 (95% CI 0.80–0.98), noninferior; Tanzania, RR 1.07 (95% CI 0.95–1.21), noninferiority not met.	Context-specific variability; adherence/logistics differed between sites	Supports the potential use of a lower calcium dose for preeclampsia prevention, although findings for preterm birth varied between settings.
Hofmeyr et al. [20]	Multicountry double-blind RCT	Women with prior preeclampsia intending pregnancy	Calcium 500 mg preconception–20 weeks vs placebo	Recurrent preeclampsia	23% vs 29%; RR 0.80 (95% CI 0.61–1.06), not significant	High-adherence subgroup: RR 0.66 (P=0.037)	No statistically significant overall reduction was observed; adherence may have influenced the magnitude of benefit.
WHO ACTION-I Trial [21]	Multicountry placebo-controlled RCT in low-resource hospitals	Women 26–33+6 weeks at risk of imminent preterm birth	Antenatal dexamethasone vs placebo	Neonatal death	19.6% vs 23.5%; RR ~0.84; composite death/stillbirth also reduced	No increase in maternal infection; benefit dependent on facility readiness	Demonstrated neonatal survival benefit in appropriately resourced healthcare settings.
Poston et al. [22]	Multicenter RCT (UK)	Obese pregnant women (BMI ≥30)	Behavioral diet + PA program vs standard care	GDM; LGA infants	No significant reduction in GDM or LGA	Improved diet quality, physical activity, and modest GWG differences	The intervention improved selected behavioral measures but did not significantly reduce the primary GDM or LGA outcomes.
Simmons et al. [23]	European multicenter pilot RCT	Overweight/obese pregnant women (BMI ≥29)	HE vs PA vs HE+PA coaching	Maternal glucose & GWG	HE vs PA: lower fasting glucose (−0.3 mmol/L; 95% CI −0.4 to −0.1; P=0.01) and lower GWG (−2.6 kg; 95% CI −4.9 to −0.2; P=0.03)	Combined HE+PA arm did not provide additional benefit	Healthy eating showed greater improvement than physical activity alone for fasting glucose and gestational weight gain, while the combined intervention did not demonstrate additional significant benefit.
Kunath et al. [24]	Cluster RCT embedded in routine care	2286 women across BMI categories	Structured counselling (4 sessions) vs usual care	Excess GWG; GDM	GWG: 45.1% vs 45.7%; GDM: 10.8% vs 11.1% (NS)	High external validity; relatively low intervention intensity	The structured counselling intervention did not significantly reduce excessive gestational weight gain or GDM compared with usual care.
Assaf-Balut et al. [25]	Single-center RCT	Normoglycemic women at 8–12 weeks	Mediterranean diet + EVOO + pistachios vs low-fat standard diet	GDM incidence	17.1% vs 23.4%; RR 0.73; adjusted RR 0.75	Also reduced insulin-treated GDM, prematurity, CS, SGA/LGA	Supports a potential preventive benefit of a structured Mediterranean dietary pattern for GDM.
Xu et al. [26]	Single-centre RCT (China)	251 women at high risk for GDM	Intensive lifestyle program vs routine care	GDM; PIH	GDM: 16.4% vs 30.9% (−46.9%, P=0.007); PIH reduced by 74.2%	No effect on CS, macrosomia, PTB	The intensive lifestyle programme was associated with lower GDM and PIH incidence in this high-risk population.
Fernandez Turienzo et al. [27]	Hybrid implementation-effectiveness pilot RCT (UK)	Women at increased risk of preterm birth	Midwifery continuity model vs standard care	Composite timely intervention outcome	83.3% vs 84.7%; RR 0.98 (NS)	Improved breastfeeding initiation and skin-to-skin contact	The model improved selected patient-centred and care-process outcomes but did not significantly alter the primary clinical outcome.
Sujan et al. [28]	Single-centre RCT (Norway)	167 high-risk individuals planning pregnancy	Time-restricted eating + exercise vs standard care	2-h OGTT glucose at 28 weeks	Mean difference 0.48 mmol/L (95% CI −0.05 to 1.01), P=0.08	Adherence declined during pregnancy; modest weight/fat differences	The intervention did not significantly improve the primary glycemic outcome, and declining adherence may have influenced its effectiveness.

Risk of Bias

Risk-of-bias assessments varied across the included randomized trials. Pharmacological trials generally showed fewer methodological concerns, although individual domain judgments differed between studies. Lifestyle and behavioral trials more frequently raised concerns related to deviations from intended interventions, adherence, missing outcome data, and outcome measurement [22-28]. All five RoB 2 domains, including selection of the reported result, were assessed, and overall judgments were derived using the RoB 2 decision algorithm. Detailed domain-level judgments are presented in Table 3.

**Table 3:** Risk of Bias Assessment (RoB 2 Tool)

Study	Randomization Process	Deviations from Intended Interventions	Missing Outcome Data	Measurement of Outcome	Overall Risk of Bias
Rolnik et al. [18]	Low risk	Low risk	Low risk	Low risk	Low risk
Dwarkanath et al. [19]	Low risk	Low risk	Low risk	Low risk	Low risk
Hofmeyr et al. [20]	Low risk	Low risk	Some concerns	Low risk	Some concerns
WHO ACTION-I Trial [21]	Low risk	Low risk	Low risk	Low risk	Low risk
Poston et al. [22]	Low risk	High risk	Low risk	Some concerns	Some concerns
Simmons et al. [23]	Low risk	Some concerns	Some concerns	Some concerns	Some concerns
Kunath et al. [24]	Some concerns	Some concerns	Some concerns	Some concerns	Some concerns
Assaf-Balut et al. [25]	Low risk	Some concerns	Low risk	Low risk	Some concerns
Xu et al. [26]	Some concerns	Some concerns	Low risk	Some concerns	Some concerns
Fernandez Turienzo et al. [27]	Low risk	Some concerns	Low risk	Some concerns	Some concerns
Sujan et al. [28]	Low risk	Some concerns	Some concerns	Low risk	Some concerns

The domain-level risk-of-bias assessment is presented in Figure 2, with overall judgments categorized as low risk, some concerns, or high risk according to the RoB 2 decision algorithm.

**Figure 2:**
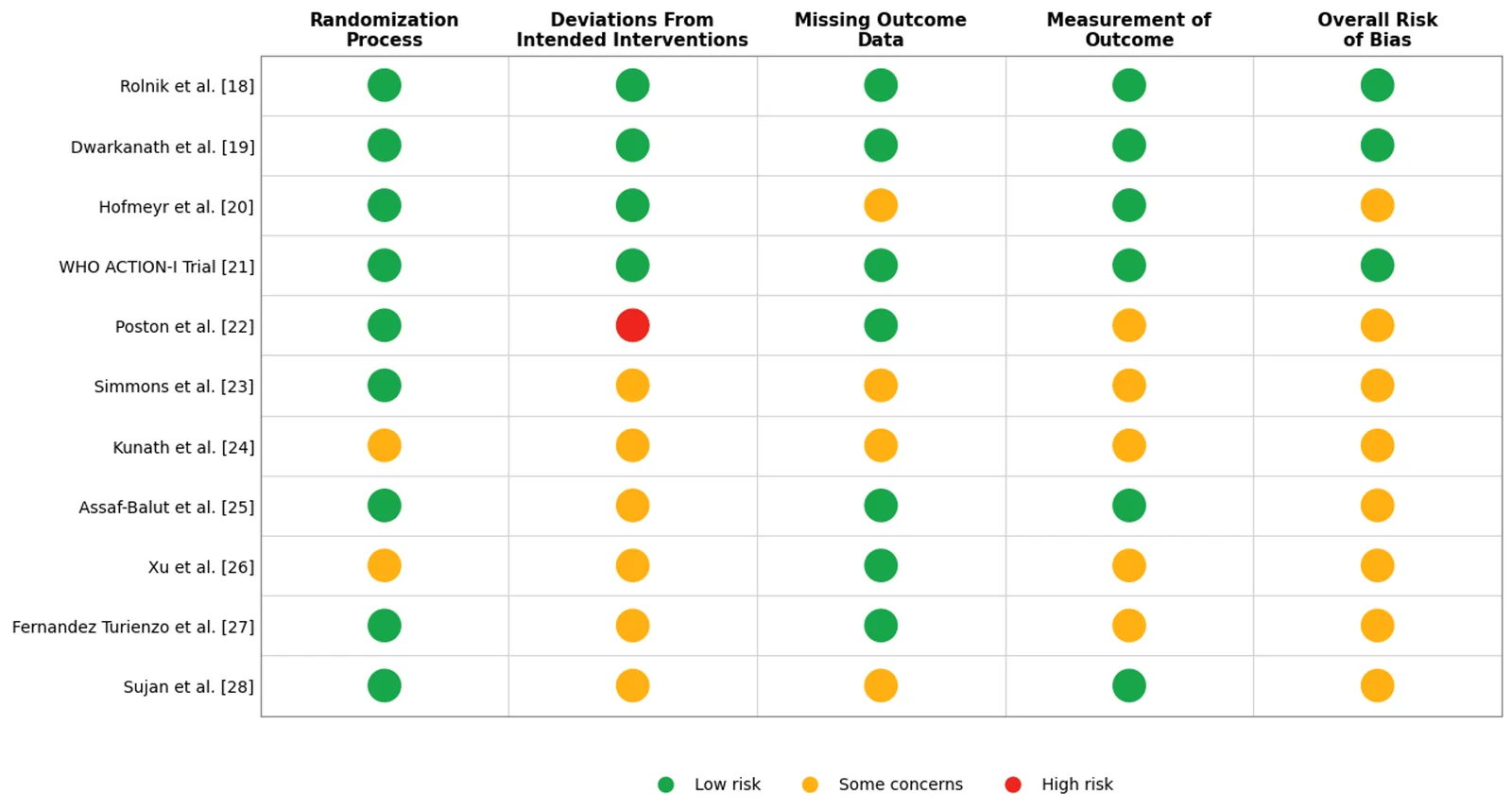
Risk of Bias Traffic-Light Plot of the Included Studies

Synthesis of Findings by Intervention Category

Studies were synthesized within prespecified intervention categories rather than pooled across categories because the interventions addressed different clinical targets, populations, and outcomes. Within each category, the direction and magnitude of reported effects, confidence intervals or other measures of precision, intervention characteristics, and risk-of-bias judgments were considered together. Findings across intervention categories were not treated as direct comparative estimates of relative effectiveness.

*Pharmacological Interventions* 

The pharmacological trials reported beneficial effects for selected predefined outcomes, although the magnitude and clinical context of these effects differed across interventions and populations. In women identified as being at high risk for preterm preeclampsia, aspirin 150 mg daily initiated at 11-14 weeks of gestation significantly reduced the incidence of preterm preeclampsia compared with placebo [18]. In the calcium supplementation trials, 500 mg/day calcium was found to be noninferior to 1500 mg/day for the prevention of preeclampsia in both India and Tanzania, although findings for preterm birth differed between the two settings [19]. In a separate trial among women with a history of preeclampsia, calcium supplementation initiated before conception and continued during early pregnancy produced a lower incidence of recurrent preeclampsia, although the overall difference was not statistically significant; a greater benefit was observed among women with higher adherence [20]. Antenatal dexamethasone administered to women at risk of imminent early preterm birth in appropriately resourced hospitals significantly reduced neonatal mortality without increasing maternal bacterial infection [21]. Overall, the findings differed according to the intervention, clinical indication, timing of initiation, dose, adherence, and healthcare setting.

*Lifestyle and Dietary Interventions* 

Lifestyle and dietary interventions produced heterogeneous findings across the included trials. A structured Mediterranean dietary intervention was associated with a lower incidence of gestational diabetes mellitus, while an intensive lifestyle program in women at high risk for GDM was associated with reductions in GDM and pregnancy-induced hypertension [25,26]. In contrast, broader behavioral interventions and routine-care counselling did not significantly improve several major clinical outcomes [22,24]. In one trial, healthy eating was associated with lower fasting glucose and gestational weight gain than physical activity alone, while the combined intervention did not show an additional statistically significant benefit [23]. A preconception time-restricted eating and exercise intervention also did not significantly improve the primary glycemic outcome [28].

Behavioral and Health System Interventions

Behavioral and healthcare-system interventions primarily influenced metabolic, care-process, and patient-centered outcomes. One trial reported lower fasting glucose and gestational weight gain with healthy eating compared with physical activity alone [23]. A midwifery continuity-of-care model improved selected care-process outcomes, including breastfeeding initiation and skin-to-skin contact, but did not significantly alter its primary composite outcome [27]. These findings indicate variation according to the type and intended target of the intervention.

Discussion

The findings of this systematic review indicate that the effectiveness of preventive interventions for pregnancy-related complications varies according to the clinical target, baseline maternal risk, timing of initiation, intervention intensity, adherence, and healthcare setting. Pharmacological interventions showed comparatively consistent benefits for selected indications, but these findings should not be interpreted as evidence that pharmacological approaches are universally superior to lifestyle, dietary, behavioral, or healthcare-system interventions because these strategies address different outcomes. Low-dose aspirin demonstrated benefit in women identified as being at increased risk of preterm preeclampsia [18], while calcium supplementation produced more context-dependent findings across dosing strategies, populations, and timing of initiation [20,29]. Antenatal dexamethasone reduced neonatal mortality among women at risk of imminent early preterm birth when administered in appropriately resourced hospitals [21]. Observational evidence has also examined the relationship between antenatal corticosteroid exposure and perinatal mortality [30]; however, such findings were considered supportive rather than equivalent to randomized trial evidence. These results support targeted use of pharmacological interventions according to the specific clinical indication, maternal risk profile, and healthcare context rather than broad assumptions of comparative superiority [31].

Lifestyle and dietary interventions demonstrated greater heterogeneity in their effects. Structured dietary approaches and intensive lifestyle programs were associated with favorable metabolic outcomes in selected populations [25,26], whereas broader or lower-intensity interventions did not consistently improve major clinical endpoints [22,24]. Healthy eating appeared to provide greater benefit than physical activity alone for selected measures of fasting glucose and gestational weight gain, while combining healthy eating with physical activity did not demonstrate additional significant benefit in the relevant trial [32]. Differences in baseline metabolic risk, intervention intensity, timing of initiation, adherence, and population characteristics may partly explain the variability between studies. Contemporary systematic-review evidence similarly indicates that the effects of lifestyle interventions during pregnancy differ across populations and intervention designs [13,33]. These findings suggest that lifestyle strategies may be more clinically useful when they are structured, sufficiently intensive, and tailored to defined risk groups rather than delivered as uniform counselling interventions. Behavioral and healthcare-system interventions appeared to influence care processes and patient-centered outcomes more consistently than major maternal or neonatal clinical endpoints. Continuity-of-care models were associated with improvements in selected aspects of maternity care, including breastfeeding-related outcomes and patient experience, while effects on preterm birth and other major clinical outcomes were less consistent [27,34]. Similar findings were observed with integrated maternity-care pathways, which altered patterns of healthcare utilization without producing clear improvements in neonatal outcomes [35]. This distinction is clinically important because improvement in continuity, accessibility, or patient experience should not automatically be interpreted as a reduction in pregnancy-related morbidity. Healthcare-system interventions may therefore be most appropriately considered as components of broader integrated care strategies rather than substitutes for condition-specific preventive measures.

Several sources of heterogeneity limited direct comparison across the included trials. Study populations differed in baseline maternal risk, body mass index, previous pregnancy complications, and metabolic characteristics. Interventions also varied in timing, dose or intensity, duration, comparator groups, adherence, and outcome definitions. Some interventions were initiated before conception, whereas others began during early or later pregnancy, further limiting direct comparability. Healthcare infrastructure also influenced the applicability of specific interventions, particularly antenatal corticosteroids, for which accurate assessment of imminent preterm birth and access to appropriate maternal and neonatal care are important [21]. Adherence was especially relevant to lifestyle and behavioral interventions, in which sustained participation may influence treatment effects [15,28,36]. These differences support interpretation of each intervention within its specific clinical and population context rather than direct ranking across intervention categories.

The findings also have implications for clinical applicability. Preventive strategies should be selected according to the complication being targeted rather than applied as interchangeable approaches. Low-dose aspirin is relevant to women at increased risk of preeclampsia [18], calcium supplementation may have a role in selected populations depending on baseline risk, dietary calcium intake, dose, and adherence [19,20], and antenatal corticosteroids are applicable to women at risk of imminent preterm birth in settings capable of providing appropriate obstetric and neonatal care [21]. Lifestyle and dietary strategies may be more appropriate for women at increased metabolic risk when programs are sufficiently intensive and adherence can be sustained [23,25]. Behavioral and healthcare-system interventions may complement these approaches by improving continuity, engagement, and selected care-process outcomes [27,34]. Clinical interpretation should therefore focus on matching the intervention to the relevant maternal or neonatal risk rather than assuming that one intervention category is broadly more effective than another. The strength of the evidence was interpreted according to study design and methodological quality. Randomized controlled trials constituted the primary evidence base of this systematic review. Observational studies were used only for contextual or supportive interpretation, while systematic reviews and meta-analyses were used to assess consistency with the broader evidence base. These sources were not treated as equivalent levels of evidence. Methodological limitations within the included trials, including difficulties with participant blinding in behavioral interventions, variation in adherence, missing outcome data, and pilot or feasibility designs, also require cautious interpretation of the findings [27,28]. The absence of quantitative meta-analysis further limits formal comparison of intervention effects because the included trials evaluated heterogeneous populations, interventions, comparators, and outcomes.

The overall evidence therefore supports a context-specific approach to prevention of pregnancy-related complications. Pharmacological interventions demonstrated benefits for selected clinical indications, while lifestyle and dietary approaches produced more variable findings that appeared to depend substantially on intensity, adherence, and population characteristics. Behavioral and healthcare-system interventions contributed mainly to care processes and patient-centered outcomes. The heterogeneity of clinical targets and study designs means that these intervention categories should not be interpreted as directly comparable. Further well-designed randomized trials are needed to clarify optimal timing, intervention intensity, adherence strategies, scalability, and the durability of maternal and neonatal benefits across diverse healthcare settings. 

Limitations and Future Recommendations

This review has several limitations. Direct comparison across studies was restricted by heterogeneity in participant characteristics, intervention type, timing, intensity, adherence, comparator groups, and outcome measures. Several trials had moderate risk-of-bias concerns related to blinding, missing outcome data, or pilot and feasibility designs. The absence of quantitative synthesis prevented estimation of pooled intervention effects, and differences in healthcare infrastructure may limit the generalizability of findings across settings. Variations in database indexing, terminology, and search syntax may also have influenced study retrieval despite the use of a structured search strategy. The review protocol was not prospectively registered, which limits the ability to verify whether all methodological decisions were prespecified. Although screening, extraction, and risk-of-bias assessment were conducted using predefined procedures, reproducibility also depends on transparent reporting of the complete database-specific search strategies and reviewer procedures. The narrative synthesis did not generate pooled effect estimates and therefore cannot establish comparative effectiveness between intervention categories. Differences in outcome definitions, effect measures, populations, and intervention designs limited direct quantitative comparison, and narrative interpretation may remain susceptible to subjective judgment despite the use of a structured synthesis approach.

Future research should prioritize standardized intervention protocols, clearly defined clinical outcomes, and adequately powered randomized trials across diverse populations. Greater attention should be given to preconception and early-pregnancy interventions, strategies to improve long-term adherence, and assessment of scalability and sustainability in routine clinical practice. Trials evaluating integrated pharmacological, lifestyle, and healthcare-system approaches may also help clarify how different preventive strategies can be combined within specific clinical contexts.

## Conclusions

The available evidence indicates that preventive interventions provide different benefits according to the pregnancy-related outcome being targeted and the characteristics of the population receiving the intervention. Low-dose aspirin, calcium supplementation, and antenatal corticosteroids demonstrated benefits for selected outcomes in defined clinical populations and settings, although their effects varied according to timing, dose, baseline risk, adherence, and healthcare capacity. Lifestyle and dietary interventions produced more heterogeneous findings, with structured and intensive approaches showing favorable outcomes in some populations while lower-intensity interventions had less consistent effects on major clinical endpoints. Behavioral and healthcare-system interventions primarily improved selected aspects of care delivery and patient-centered outcomes, with less consistent effects on major maternal and neonatal outcomes. Because the included interventions addressed different clinical targets and were evaluated across heterogeneous populations, settings, and outcomes, direct ranking of their relative effectiveness is not warranted. These findings support targeted and context-specific preventive strategies and highlight the need for well-designed trials addressing intervention timing, adherence, scalability, and clinically relevant maternal and neonatal outcomes.
